# Ultrasound-Assisted Community Bureau of Reference (BCR) Procedure for Heavy Metal Removal in Sewage Sludge

**DOI:** 10.3390/ma17225452

**Published:** 2024-11-08

**Authors:** Nicoleta Mirela Marin, Toma Galaon, Luoana Florentina Pascu

**Affiliations:** 1 National Research and Development Institute for Industrial Ecology-ECOIND, 57-73 Drumul Podu Dambovitei, 060652 Bucharest, Romania; nicoleta.marin@incdecoind.ro; 2 Department of Oxide Materials Science and Engineering, National University of Science and Technology Politehnica Bucharest, 1–7 Gh. Polizu, 060042 Bucharest, Romania; 3 Department of Analytical and Physical Chemistry, University of Bucharest, 4-12 Regina Elisabeta Bd., 030018 Bucharest, Romania

**Keywords:** ultrasound-assisted BCR extraction, sewage sludge, leachate, maize stalk, metal removal

## Abstract

Sewage sludge (SS) resulting from wastewater treatment plants (WWTP) is commonly applied worldwide as a fertilizer in agriculture. This can be done following a rigorous analysis of the sewage sludge composition. Due to its toxic potential, heavy metal ion content is one of the key parameters to test when evaluating SS sample usage as fertilizer. The distribution of metals present in SS samples produced by five municipal WWTPs in Romania was studied. To obtain information regarding metal distribution in SS, a modified ultrasound-assisted extraction procedure of the Community Bureau of Reference (BCR) was employed for As, Cd, Cr, Cu, Mo, Ni, Pb, Zn, and Co quantitation. Concentrations of these metals were measured using ICP-EOS spectrometry. Method extraction accuracy was verified using CRM-483 certified reference material. Results show that extraction efficiency was lowest for the exchangeable fraction for all studied metals. The detected ion metals were found distributed in fractions (F) 2, 3 and 4, which are unavailable for plants and groundwater under natural environmental conditions. One noteworthy finding was that using ultrapure water for the leachate test resulted in low metal solubility, indicating slight metal desorption in real environmental samples. Furthermore, maize stalk bio-adsorbent was used to minimize metal ion content in WWTP leachate samples produced by the storage of SS in terms of metal ion adsorption.

## 1. Introduction

The treatment and disposal of sewage sludge are critical components of wastewater management, aiming to minimize environmental impact while maximizing resource recovery [1,2,3,4]. Several technological processes are involved in sewage sludge treatment. Aerobic stabilization uses aerobic microorganisms to break down organic matter in SS [5]. This usually requires aeration to maintain oxygen levels, resulting in a reduction of pathogens and odors while converting organic material into a more stable form. Anaerobic stabilization uses microorganisms that decompose the organic matter without oxygen, producing biogas as a by-product [6]. This process not only stabilizes the SS but also generates renewable energy. In the final step, dehydration is applied. After SS stabilization, water is released to obtain a solid content up to 20–30% dry matter [7]. Techniques such as centrifugation, belt presses, or filter presses are commonly used. Dehydration reduces volume and prepares the SS for further processing or utilization. The treated SS contains valuable nutrients like nitrogen, phosphorus, and organic matter that can enhance soil fertility and structure. When adequately processed, SS can be used as a soil amendment or fertilizer, promoting plant growth and improving soil health. Despite its potential benefits, direct land application of SS presents several challenges: SS may contain metals, pharmaceutical residues, pathogens, and other toxic compounds that can present risks to human and environmental health [8,9]. Today, monitoring and strict regulations are crucial to ensure that the levels of these contaminants fall within safe limits. However, addressing the challenges posed by toxic substances and ensuring public safety are paramount in promoting the sustainable and healthy use of treated SS. Ongoing advancements in treatment technology and equitable regulatory measures will contribute to overcoming these challenges and enhancing the overall efficacy of SS management [1,10].

The assessment of heavy metal content in waste materials, particularly in the context of their use for agricultural purposes, is critical for understanding their potential environmental impact. Total content of metals does not provide a complete image of metals that can interact with the soil or be taken up by plants.

Instead, the bioavailability of these metals needs to be evaluated, which involves understanding the fractions to which they are bound within the material. The sequential extraction method has been widely used for this purpose [11,12,13,14,15]. This method allows for the categorization of metals into distinct fractions, which can indicate their mobility and potential bioavailability.

The fractions defined by this method include exchangeable fractions where the metals that are loosely attached to the SS particles are more readily available for uptake by plants or leaching into groundwater together with an acid soluble fraction when metals are associated in carbonate forms (F1). This fraction can be released under changes in pH, making them bioavailable under certain conditions. In the reducible fraction, metals are complexed with oxides or hydroxides of Fe and Mn (F2). These can be released under reducing conditions, further indicating their potential mobility. The oxidizable fraction consists of metals bound to organic matter, which can be released upon the decomposition of organic material or changes in redox conditions (F3). In the residual fraction are found metals that are strongly bound to the mineral matrix and are less likely to be mobile or bioavailable (F4). Understanding the binding mechanisms of metals in SS is vital for making informed decisions regarding its application in agriculture.

Also, in general, in-situ technologies offer a significant advantage as they are cost-effective and eliminate the need for additional transportation and handling costs. However, the SS produced by WWTPs is stored, leading to the leaching of dissolved compounds containing significant concentrations of soluble metals. For agricultural SS, it is essential to utilize the dried mass, despite the substantial amounts of leachate generated. Furthermore, in addition to conventional leachate treatment technologies, the control of metal ion retention from the liquid on the solid phase surface can be effectively managed through cation exchange and the adsorption process. Cellulose is a natural polymer found in the cell walls of green plants. It is an essential macro-component for maintaining the structure of plants. Cellulose has a linear structure made up of glucose residues and has a low capacity to exchange ions [16,17,18,19,20].

As part of this study, samples of leachate generated by the storage tank were collected from each municipal wastewater treatment plant. In the experimental phase of this study, the aims were to develop analytical methods for the detection of the As, Cd, Cr, Cu, Mo, Ni, Pb, Zn, and Co by using inductively coupled plasma optical emission spectrometry (ICP-OES). Our tests assess the mobility of metals in SS from urban wastewater treatment plants. To quantify metal concentrations, we used reagents of varying aggressivity to selectively analyze different phases, using water for soluble forms, which is essential for assessing the risk of groundwater and surface water contamination, and weak complexing/extraction agents to assess accessibility of metals to plants. In this part of the study, the modified BCR extraction method was used by reducing the time of extraction from 16 h when mechanical stirring is used to only 20 min using ultrasonic extraction at 50 kHz frequency and T = 25 ± 2 °C. Activated cellulose can be used as an excellent ion exchanger to concentrate and separate ions in the function of pH. In working towards implementing a new environmentally friendly technology, it is important to consider the potential of maize stalk-based vegetable waste as an alternative to traditional ion exchange resins. Taking into consideration this aspect, a green novel technology was used to minimize metal ion content in SS leachate samples.

## 2. Materials and Methods

### 2.1. Reagent

To ensure the quality of the analytical determinations, all reagents were of analytical purity. The metal standard solution of certified reference material (CRM) consisted of 1000 mg/L As, Be, Cd, Co, Cr, Co, Cu, Fe, Pb, Li, Mg, Mn, Mo, Ni, Sb, Se, Sr, Tl, Ti, V, and Zn and were purchased from Merck (Darmstadt, Germany). The reagents used included CH_3_COOH (glacial), density 1.049 g/mL, HNO_3_ density 1.4 g/mL, HCl density 1.16 g/mL, NH_2_OH-HCl, CH₃COONH₄ 98% purity, and hydrogen peroxide (H_2_O_2_) 30% (*w*/*w*) in H_2_O (Merck).

### 2.2. Equipment

For metal determination, an inductively coupled plasma optical emission spectrometer Optima 5300 DV (ICP-OES, Perkin Elmer, Norwalk, CT, USA) equipment was used. The grinding mill (PM 100, Retsch®, Haan, Germany) was employed for grinding dried SS samples, the vibrating sieving system (Analysette 3 PRO, Frisch GmbH, Idar-Oberstein, Germany) with mesh size less than 63 μm was used for sieving dried sewage sludge, and a horizontal rotary shaker (GFL 3017, GFL; Burgwedel, Germany), analytical balance with a precision of ± 0.0001 g (XT220A Precisa, Dietikon, Switzerland), electric hotplate (ROTILABO^®^ MH 15, Carl Roth, Karlsruhe, Germany), sand bath, Milestone Ethos Up microwave digester (Milestone Srl, Sorisole, Italy), and a centrifuge (Universal 320, Andreas Hettich GmbH & Co. KG, Tuttlingen, Germany) applying a speed range 0–5000 rpm for 30 min, were employed for SS analysis; also, a pH meter (HI 255 Hanna Instruments, Nijverheidslaan, Belgium) was used to adjust the pH of the extraction solutions. 

### 2.3. Exchangeable and Acid Solution

To obtain the 0.43 M CH_3_COOH solution at pH = 2.5, the following steps were taken into consideration. Initially, in 800 mL of ultrapure water, 12.3 mL of CH_3_COOH were added. Subsequently, the solution was mixed with a magnetic stirrer, and additional CH_3_COOH was added in drops until the desired pH was achieved. Finally, the solution was quantitatively transferred to a 1000 mL volumetric flask and filled to the mark with ultrapure water.

### 2.4. Reducible Solution

To prepare the 0.1M NH_2_OH-HCl solution, 6.95 g of NH_2_OH-HCl were dissolved in 900 mL ultrapure water. The solution was then acidified with HNO_3_ until a pH = 2 was obtained. Next, the solution was transferred to a 1000 mL volumetric flask and made up to the mark with ultrapure water.

### 2.5. Oxidable Solution

The 1M ammonium acetate solution was prepared by weighing 77.08 g of CH_3_COONH_4_ on an analytical balance and adding it to a Berzelius flask. Ultrapure water was then added and mixed until the salts were completely dissolved. The resulting solution was transferred to a 1000 mL Berzelius glass. Next, the solution was diluted with 800 mL of ultrapure water, and the pH was adjusted to a value of 2 using a few drops of concentrated HNO_3_. Finally, the solution was transferred into a 1000 mL volumetric flask and diluted to the mark with ultrapure water.

### 2.6. Collected SS and Leachate Samples

For SS evaluation, a representative sample was collected from each WWTP. These samples underwent a series of conditioning processes, including drying, grinding, and sieving, before analysis. The SS samples were collected in plastic recipients and allowed to air-dry on plastic trays at room temperature. After 8 days of air drying, each sample was crushed using a mortar and sieved through a nylon sieve with a mesh size less than 63 μm at 25 ± 2 °C.

Also, a representative sample of leachate generated from the landfill SS storage was taken from each wastewater treatment plant.

### 2.7. Application of Chemical Extraction Steps

In this part of the study, the modified BCR method was used by reducing the time of extraction from 16 h to 20 min using ultrasonic agitation at 50 kHz and 25 ± 2 °C. Ultrasonic baths make use of cavitation bubbles created by high-frequency pressure (sound) waves to stir a liquid/solid phase. Ranging from 25 to 80 kHz, the ultrasound waves create millions of imploding bubbles, leading to dissolution and homogenization of different chemical species from the solid to liquid phase.

Step 1 Over 1 g of dried SS with a particle size of 63 μm, 20 mL of CH_3_COOH 0.11M (pH = 2.8) was added, and the resulting solution was sonicated in the ultrasonic bath for 20 min at 50 kHz and 25 ± 2 °C, and centrifuged at 5000 rpm for 30 min, and the decanted supernatant was analyzed by ICP-OES, F1. The samples were washed with 20 mL of ultrapure water between each extraction step.
Step 2 The residue obtained from the first step of F1 was agitated for 20 min at 50 kHz at 25 ± 2 °C using 20 mL of NH_2_OH-HCl (pH = 2). After 30 min of centrifugation at 5000 rpm, the decanted supernatant was analyzed by ICP-OES, F2.
Step 3 The residue obtained from the second step was treated in an ultrasonic bath with 30% H_2_O_2_ until evaporation was done in ≈30 min. Subsequently, 20 mL of 1M CH_3_COONH_4_, pH = 2 was added over the dry residue, and the samples were stirred for 20 min, at 50 kHz and 25 ± 2 °C. The supernatant obtained after 30 min of centrifugation at 5000rpm was analyzed by ICP-OES, F3.
Step 4 The residue obtained from the F3 was washed with 20 mL of ultrapure water and treated for 30 min in a microwave oven with aqua regia (HCl: HNO_3_ in a 3:1 ratio 21 mL HCl: 7 mL HNO_3_) added to determine the residual fraction, F4.

### 2.8. Metal Analysis

The ICP-OES method was used to analyze the dissolved metal ions in the liquid phase and in the total metal content for the solid part of SS taken from municipal WWTPs.

The principle of the ICP-OES spectrometric method is based on the generation of an aerosol from the analyzed solution, an aerosol which is then transported to the plasma torch for atomic excitation. The inductively coupled plasma creates atomic emission spectra, which are characteristic for each metal. The peak height/area of each detected metal is then further corelated with its concentration using a linear regression calibration curve.

### 2.9. Determination of Total Metal Content in SS Samples

The metal content of the SS samples was determined using aqua regia metal extraction procedures. For this, 6 mL of HCl and 2 mL of HNO_3_ were added to 1 g of dry SS samples. The samples were then weighed and placed in the containers of the microwave digester Milestone Ethos Up. Subsequently, a digestion program in 3 steps was employed to ensure maximum extraction. The digestion program included first heating the liquid/solid phase to 220 °C for 15 min, followed by a constant temperature phase of ≈20 min, and finally a cooling phase of about 30 min was included. After digestion, the samples were filtered and transferred into volumetric flasks. The total metal content was detected using the ICP-OES method.

### 2.10. Leaching Test Applied to SS Samples

The ability of SS samples to transfer metal ions to the environment (soil, plants, surface water, and groundwater) was evaluated using the leaching test 1–10, SS-ultrapure water (mass/volume), according to the SR EN ISO 12457 standard—1:2003 [21].

The leaching test was applied for the quantitative determination of metal mobile content using ICP-OES. SS samples were collected, dried, and sieved, and particles less than 63 µm were subjected to the leaching test in a ratio of 1:10 (mass: volume) being SS: ultrapure water.

The mobility of metals in solution was evaluated on 5 g of dried SS from each set of collected samples. These were quantitatively placed in 100 mL Erlenmeyer flasks, over which 50 mL of ultrapure water was added. The samples were stirred for 24 h at a stirring speed of 30 rpm at 25 ± 2 °C. The extraction of metals in aqueous solution was carried out in the aqueous environment at a pH that varied between 7.0–8.5. After stirring, the obtained suspension was left in standby for 10 min to separate the solid granules. The leachate obtained was centrifuged for 30 min at 5000 rpm and passed through a 0.45 µm filter. The liquid samples obtained were subjected to analysis conducted using ICP-OES.

### 2.11. Methodology Used to Retain Metal Ions on Maize Stalks from Leachate Samples

Over 0.5 g of activated maize stalk, 50 mL of leachate samples (S1–S5), pH ≈ 8.5 was added in a 100 mL Erlenmeyer flask, the mixtures obtained were subjected to mechanical agitation for 60 min. At the end of stirring, each sample was centrifuged for 30 min at 5000 rpm, the supernatant obtained was filtered, and the concentration of metal ions that was not retained on the maize stalk was determined with the ICP-EOS. For the shredding and activation of the maize stalk, the same procedures as presented in our previous studies [17]. The percentages of metals removed from the leachate samples by shredded maize stalk were determined by applying Equation (1):

(1) $$R(\%)=\frac{\left(C_{i}-C_{e}\right)}{C_{i}}\times 100$$

where *C_i_* (mg/L) and *C_e_* (mg/L) are the initial and equilibrium concentration metals in leachate samples.

## 3. Results and Discussion

### 3.1. Evaluation of the Metallic Mobile Fraction from SS Samples

Investigating the mobility of metals in SS obtained from WWTPs is crucial to establish the final destination of the respective sewage sludge [22]. In this study, we proposed a rapid extraction method to determine the mobile metal available in different fractions. This will provide a complete overview of the metal distribution in the SS structure together with the chemical procedures for determining the total and leachable content. The sequential chemical extraction procedure is a widely used evaluation method that provides information on environmental pollution when SS is stored or used as an agricultural fertilizer. By using selective extractants, this procedure can mobilize different metal species available over time and provide information on the behavior of pollutants in common environmental conditions. In this study, the total content of metals and their species in different SS samples from five WWTPs was investigated. Our results will enable policy makers to make informed decisions on the safe disposal and management of SS to ensure a healthier and safer environment when managing the SS from WWTPs.

For the exchangeable fraction (F1), we extracted the metals soluble in 0.11M CH_3_COOH at pH 2.8. This fraction can provide valuable insights into which metals are mobile for vegetation when using SS as an agricultural fertilizer.

The reducible fraction F2, also known as the reducible fraction, plays a crucial role in evaluating the metal content bonded to Fe and Mn oxides and hydroxides. These compounds are released during the extraction process that involves a reduction potential. To create the ideal conditions for the metal fraction, NH_2_OH-HCl is the reagent that was chosen. This fraction is vital for accurate assessment and understanding of the metal content in the SS matrix.

The oxidizable fraction F3 plays a crucial role in releasing metals associated with sulfides and organic matter. When SS is used as a soil fertilizer, the toxic metals linked to this fraction can persist in the soil for extended periods. These metals are absorbed in humic substances and are gradually released due to weathering. Utilizing procedures involving oxidation with H_2_O_2_ followed by an additional extraction step with CH_3_COONH_4_ 1M at pH = 2 can effectively prevent the reabsorption of extracted metals into the oxidized substrate. Due to the high selectivity of organic substances for divalent ions, this fraction yields the highest concentrations of metallic elements. However, it is important to note that when SS is used as an agricultural fertilizer, the toxic metal elements associated with organic matter are unavailable to become mobile for agricultural crops.

The residual fraction F4 releases metallic elements that are strongly retained in the silicate form.

In light of preserving natural resources, the quality of SS needs to comply with the criteria imposed by National [22,23] and European legislation [24]. The limit value for metal concentrations in SS for Romanian and Environmental Protection Agency (EPA) legislation is presented in Table S1 of the Supplementary Materials. If we compare the values for metals imposed by the Romanian legislation with those imposed by the EPA legislation, it can be observed that the standards in our country are much more restrictive than those imposed by the EPA in terms of maximum values for As, Cd, Cu, Ni, Pb, and Zn. In addition, a limit for Cr is not imposed by the EPA legislation. Likewise, for Mo and Sb, no values are imposed in either legislation, while a limit on Co is imposed only for the total metal content in the Romanian Legislation.

**Figure 1:**
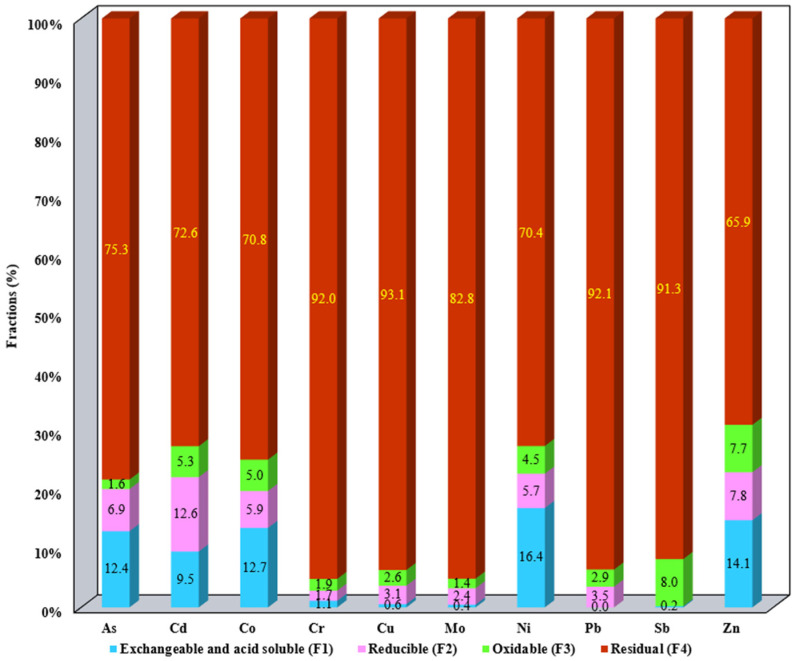
Distribution of metals in F1, F2, F3, and F4 for S1.

**Figure 2:**
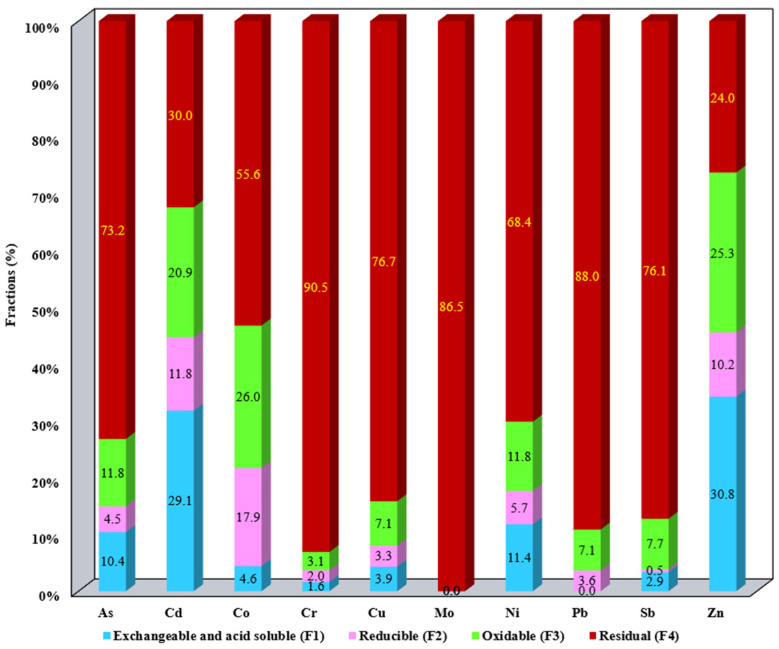
Distribution of metals in F1, F2, F3, and F4 for S2.

**Figure 3:**
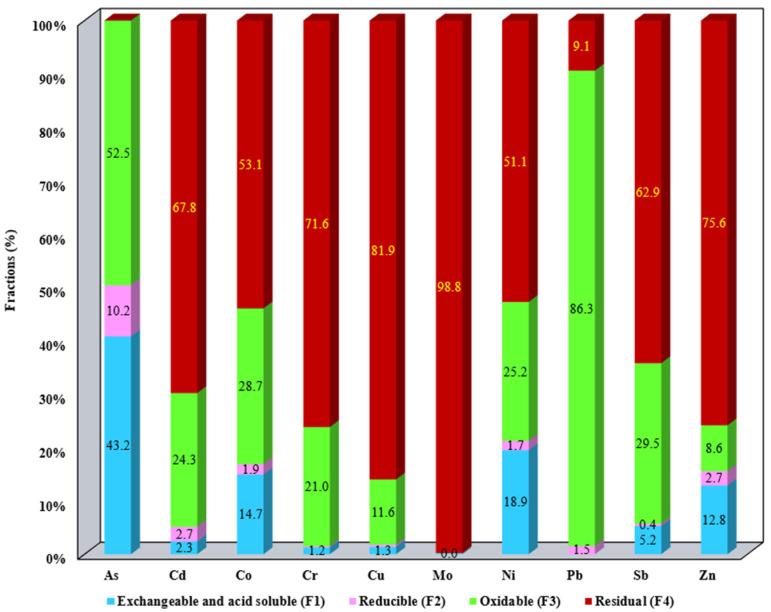
Distribution of metals in F1, F2, F3, and F4 for S3.

**Figure 4:**
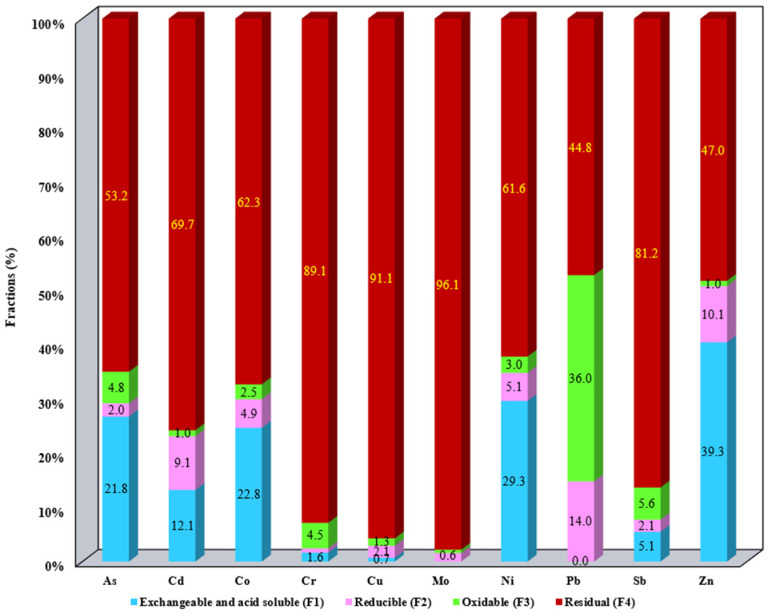
Distribution of metals in F1, F2, F3, and F4 for S4.

**Figure 5:**
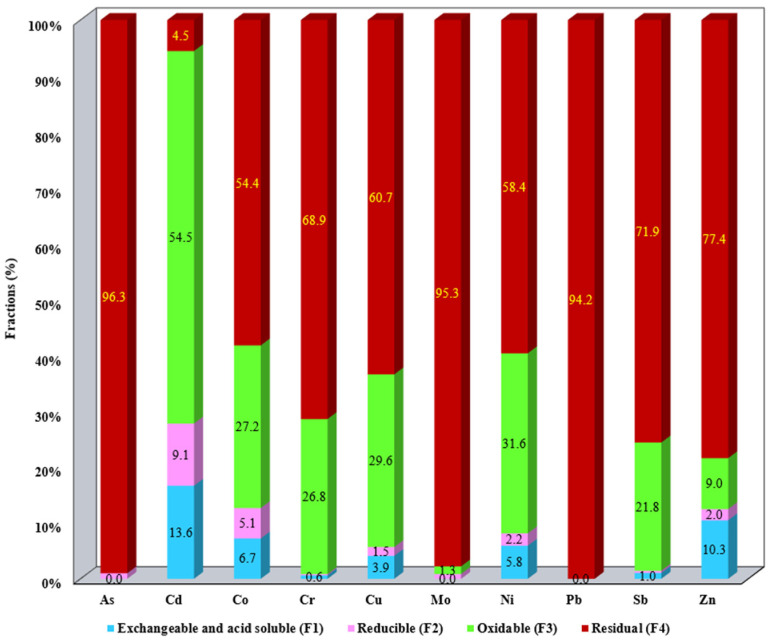
Distribution of metals in F1, F2, F3, and F4 for S5.

The results of metal fractionation distributed in the SS samples as well as the aqua regia-extractable metals are summarized in Tables S2–S6 of the Supplementary Materials. The results presented in Tables S2–S6 of the Supplementary Materials were employed for the metal mobility evaluation (Figure 1, Figure 2, Figure 3, Figure 4 and Figure 5).

Thus, for the total metal content evaluation when aqua regia is used, it was found that although the maximum concentrations were below the values imposed by the Romanian and EPA legislations, Zn was the metal with the highest values in the studied SS samples as presented in Tables S2–S6 of the Supplementary Materials.

The water-soluble leachable fraction does not release the metals tested as is presented in Table S7 of the Supplementary Materials. Based on this, the samples studied can be used as agricultural fertilizer. This is because the concentrations of As, Cd, Cr, Co, Cu, Mo, Ni, Pb, Sb, and Zn remain immobile for vegetation, tend to stay associated in the oxidizable fraction over time, and thereby do not have a negative impact on the environment.

The distribution of metals in the studied SS allows us to assess their mobility and bioavailability. The metal content for each studied fraction F1–F4 for all SS samples is presented in Figure 1, Figure 2, Figure 3, Figure 4 and Figure 5. Data presented include the order of mobility for each studied fraction.

F1 shows the order of the most mobile metals that can be released under natural environmental conditions. Thus, for S1 the most mobile metals were Ni (16.4%), Zn (14.1%), Co (12.7%), As (12.4%), and Cd (10%), respectively. Also, the reducible fraction shows a lower metal mobility than the first fraction. The rest of the toxic metals are associated with the low mobility of fractions F3 and F4, respectively, which do not represent a leachability hazard to the environment (Figure 1).

For S2, the mobility order of metals is Zn (30.8%) > Cd (29.1%) > Ni (11.4%) > As (10.4%), and the rest of the studied metals were below As values for F1. For the second fraction, the significant mobility above 10% was obtained for Co (17.9%) > Cd (11.8%) > Zn (10.2%), and the third fraction, they are Co (26%) > Zn (25.3%) > Cd (20.9%) > As and Ni (11.8%). Also, As (73.2%), Cd (30%), Co (55.6%), Cr (90.5%), Cu (76.7%), Mo (86.5%), Ni (68.4%), Pb (88%), Sb (76.1%), and Zn (24%) are metals that can be classified as practically immobile due to high percentages of those elements in the residual fraction (F4) (Figure 2).

For S3, As is distributed between the exchangeable and reducible fraction at 43.2% and 52.5%, respectively. A high mobility level of As for F1 can also be an indicator that entering soil; i.e., this model, is relevant to anthropogenic pollution outputs. Metals bound to organic matter and sulfides (F3) can be released easily in oxidizing conditions; therefore, an oxidation process utilized for leaching metals is related with this phase. The organics exhibit selectivity for divalent ions. This fraction has released metals in the following order: Pb (86.3%), As (52.5%), Sb (29.5%), Co (28.7%), Ni (25.2%), Cd (24.2%), Cr (21%), and Zn (8.6%). For the residual fraction, with the exception of As and Pb, most of the metals are bound in the residual fraction being found in percentages greater than 50% (Figure 3).

For S4, the metals are mostly distributed in the reducible fraction F4 in percentages that ranged from 44.8% for Pb to 96.1% for Mo. Insignificant percentages of metals were detected in fractions F2 and F3 with the exception of Pb that was found in proportions of 14% in F2 and 36% in F3. Also, appreciable amounts of Zn (39.3%), Co (22.8%), As (21.8%), and Cd (12.1%) were found associated with the soluble fraction S1 (Figure 4).

Fractional analysis of S5 shows that metals are mostly associated with fractions F3 and F4 (Figure 5). This behavior suggests the possibility that the metals are retained by organic matter that forms stable organometallic complexes, which significantly reduce their mobility into the environment. It can be concluded that, as a result of the investigations obtained by applying sequential chemical extraction, most of the metals are found accumulated in F2, F3, and F4 (more than in the exchangeable fraction), which have a low potential of availability for soil, vegetation, and ground water under normal environmental conditions.

### 3.2. Evaluation of the BCR Extraction Procedure by the Certified Reference Material

For the BCR reference material, 5 g of soil amended with SS, CRM 483 was utilized. The soil was thoroughly homogenized and mixed with 200 mL of extraction solution at pH 2.5 (Table 1). The resulting suspension was sonicated at 25 ± 2 °C for 20 min and 50 kHz. After sonication, the solution was centrifuged at 5000 rpm for 30 min, then filtered, and subsequently subjected to analysis using the ICP-OES spectrometer. Subsequently, the CRM sample was analyzed in triplicate for assurance of accuracy and only the mean value was added. Examining the data presented in Table 2, it can be observed that the ultrasound modified BCR extraction offers results very close with respect to the standardized method which used 16 h of stirring at 30 rpm as given in the certified reference material (CRM 483). The percentage difference (Bias %) between BCR modified extraction using sonication and BCR certificate value (using 16 h stirring) is much lower than the uncertainty of the method for Cd, Cr, Cu, Ni, and Zn. In the case of Pb, the bias value is 8.6%, which is included in the corresponding uncertainty value of 9.5%. These results indicate the fact that the ultrasound-assisted BCR method is equivalent to the classical BCR method. Using 20 min sonication instead of 16 h of mechanical stirring can generate an increase in the sample throughput of an environmental laboratory.

### 3.3. Application of Maize Stalk for Metal Ions Removal from Leachate Concentrated Samples

It is known that the pH of solution in adsorption studies is the most important parameter in order to improve the adsorption capacity of biosorbent materials [25]. Thus, biosorbents contain functional groups such as HO^−^, -COO^−^, -CO^−^, NH-, and SH-, which, in function of solution pH, are found in different forms as is presented in Equation (2) [25,26,27].

-BH^+^ ⇄ -BH ⇄ -B^−^ (2)

where -BH represents the bioadsorbent charge.

In acidic pH medium, these groups are protonated and act as positively charged species (-BH^+^). Subsequently, deprotonation of these groups appears with increasing pH, and they behave as zero charged groups (-BH). When the pH of the solution increases from acid to a slightly acidic region, the positive character is transformed into a negative one (-B^−^). Thus, ionizable groups begin to attract the positively charged metal ions in a slightly acidic medium taking into account data presented in literature [18,20,27,28,29,30,31].

The most adsorbed metal will be estimated based on the percentage of removal. In this study, the adsorption capacity of shredded maize stalks was evaluated by using the batch technique for the removal of As, Cd, Co, Cr, Cu, Mo, Ni, Pb, Sb, and Zn from leachate S1–S5. Thus, the adsorption experiments were carried out in a single step by mixing 0.5 g of shredded maize stalk with 50 mL leached solution for each treatment station S1L–S5L, pH = 8.5. First, the *C_i_*(mg/L) was determined for each sampling point, and the results obtained are presented in Table 3. As one can observe, significant concentrations from the majority of metals are presented in all leachate samples investigated. The most abundant metals in S1L–S5L were As, Co, Cu, Ni, and Zn while Cd, Cr, Mo, Pb, and Sb had the lowest concentrations.

In order to not produce secondary pollution in the aquatic environment, the concentration of these leachates was reduced by applying shredded maize stalks.

The obtained results are presented as follows: when maize stalk is employed for leachate S1L depollution, Cu and Sb were mostly retained in biosorbent masses, and their percentages were 88% for Cu (*C_i_* = 3.895 mg/L) and 77% for Sb (*C_i_* = 0.928 mg/L).

Also, in terms of percentage of removal, the affinity for individual metals studied can be arranged in the following order: 67% Ni (*C_i_* = 2.413 mg/L) > 56% As (*C_i_* = 0.685 mg/L) and Co (*C_i_* = 0.427 mg/L) > 52% Cd (*C_i_* = 0.190 mg/L) > 51% Zn (*C_i_* = 103mg/L) > 47% Pb (*C_i_* = 0.129 mg/L) > 18% Mo (*C_i_* = 0.09mg/L).

For S2L, the highest percentage was obtained for Zn (99.6%) *C_i_* = 271mg/L, As (97.9%) *C_i_* = 0.945 mg/L, Cr (95.8%) *C_i_* = 1.048 mg/L, Co (95.5%) *C_i_* =1.406 mg/L, Pb (80.9%) *C_i_* = 0.209 mg/L, Cu (65.7%) *C_i_* = 1.760 mg/L, and Ni (56.8%) *C_i_* = 9.718 mg/L, and low percentages under 50% were obtained for Sb (45.3%) *C_i_* = 1.880 mg/L, Cd (29.3%) *C_i_* = 0.174 mg/L, and Mo( 24.5%) *C_i_* = 0.106 mg/L.

For S3L, a significant adsorption was obtained for Mo (66.2%) *Ci* = 0.133 mg/L. For other metals, the retained percentages were below 50%.

Also, for S4L, values under 50% were obtained for majority of metals studied with the exception of Pb when 77.5%, *C_i_* = 0.550 mg/L was retained on the maize stalk mass.

**Figure 6:**
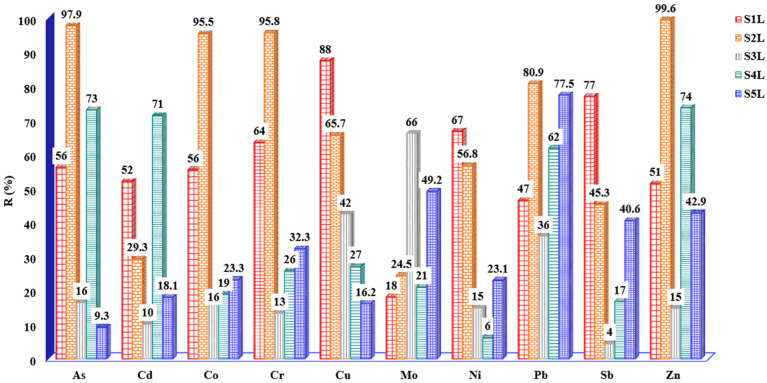
Percentage of metals removed from leachate samples on shredded maize stalk.

On the other hand, for S5L, the mostly retained metals were As (77.5%) *C_i_* = 0.626 mg/L, together with Cd (*C_i_* = 0.083 mg/L), Co (*C_i_* = 0.266mg/L), Cr (*C_i_* = 0.265 mg/L), and Cu (*C_i_* = 1.170 mg/L). All experimental results regarding percentage of removal are presented in Figure 6.

To conclude, the most retained metals from leachate samples on maize stalk masses were Cu (*C_i_* = 3.895 mg/L) for S1L, Zn (*C_i_* = 271mg/L) for S2L, Mo (*C_i_* = 0.133 mg/L) for S3L, Zn (*C_i_* = 1.730 mg/L) for S4L, and Pb (*C_i_* = 0.182 mg/L) for S5L. As presented in Figure 6, the maize stalk manifests a good capacity to remove in various percentages the majority of the investigated metals.

## 4. Conclusions

The total concentration of metals obtained from sewage sludge is not adequate in revealing the potential for toxic metals to migrate into the environment. Understanding the forms in which these metals are bound within the SS structure is crucial for assessing their toxicity.

Sequential chemical extraction procedures can be used to analyze the mobility of these metals, providing valuable information for soil fertilization techniques when utilizing SS as an agricultural fertilizer.

For the complete evaluation of the SS from the municipal WWTPs, it is necessary to analyze the total content of metals as well as the quantitation of the available metallic forms that provide information about the behavior of Cu, Pb, Ni and others over time under the influence of environmental factors.

Applying the sequential BCR extraction scheme can highlight the mechanisms that govern the bioavailability process under normal environmental conditions, as well as the distribution of metals in different available fractions using extractions with different chemical aggressiveness that are bioavailable for plants and environmental medium. So, evaluation based just on legal limit values would not be adequate to protect the entire population.

The BCR extraction procedure was modified using ultrasound (50 kHz frequency, 25 °C water temperature), leading to a significant reduction in sample preparation time from 16 h to only 20 min.

Experimental data revealed that the content of metals in the SS samples does not exceed maximum accepted values for agricultural usage. The analysis of the four fractions shows that in fractions F2, 3 and F4 the metals are bound, making them practically unavailable for plants and the aquatic environment. Thus, the tested SS samples can be used in agriculture for fertilizing purposes.

At the same time, the feasibility of using maize stalks in environmental pollution reduction processes was evaluated for the retention of metal ions from leachate produced by the storage of sewage sludge.

## Figures and Tables

**Table 1:** Conditions for evaluating the BCR procedures.

Method	Extracting Solutions	pH	Conditions	
BCR	0.11 M CH_3_COOH	2.8	Report (m/V) = 1:40, 20 min, 50 kHz, 25 ± 2 °C	Our laboratory conditions
method	0.43 M CH_3_COOH	2.5	Report (m/V) = 1:40, 16 h, 30 rpm, 25 ± 2 °C	BCR conditions from certified

**Table 2:** The determined and certified values for the reference material BCR-483.

Metal	Determined Value		Certified Value	Recovery	Bias (%)
	mg/Kg DM	Uncertainty (±%)	mg/Kg DM	(%)	
Cd	17.5	10	18.3	95.6	4.4
Cr	18.9	9	18.7	101.1	−1.1
Cu	33.9	9	33.5	101.2	−1.2
Ni	24.7	10	25.8	95.7	4.3
Pb	1.92	9.5	2.10	91.4	8.6
Zn	611	9.5	620	98.5	1.5

**Table 3:** Initial concentration of leachate samples (S1L–S5L), (Mean ± S_dev_).

Metal (mg/L)	S1L	S2L	S3L	S4L	S5L
As	0.685 ± 0.027	0.945 ± 0.034	0.620 ± 0.036	0.156 ± 0.006	0.626 ± 0.020
Cd	0.190 ± 0.024	0.174 ± 0.006	0.207 ± 0.015	0.350 ± 0.022	0.083 ± 0.004
Co	0.427 ± 0.095	1.406 ± 0.015	0.579 ± 0.013	0.390 ± 0.010	0.266 ± 0.012
Cr	0.603 ± 0.069	1.048 ± 0.013	3.185 ± 0.051	0.732 ± 0.012	0.265 ± 0.008
Cu	3.895 ± 0.006	1.760 ± 0.021	2.191 ± 0.104	6.000 ± 0.160	1.790 ± 0.0174
Mo	0.099 ± 0.024	0.106 ± 0.003	0.133 ± 0.003	0.038 ± 0.008	0.061 ± 0.004
Ni	2.413 ± 0.077	9.718 ± 0.021	4.061 ± 0.031	0.902 ± 0.004	1.300 ± 0.062
Pb	0.129 ± 0.041	0.209 ± 0.003	0.025 ± 0.003	0.550 ± 0.021	0.182 ± 0.005
Sb	0.928 ± 0.122	1.880 ± 0.039	1.220 ± 0.070	0.975 ± 0.006	0.926 ± 0.006
Zn	103 ± 0.850	271 ± 3.266	210 ± 3.742	1.730 ± 0.049	350 ± 1.247

## Data Availability

The original contributions presented in the study are included in the article and Supplementary Material, further inquiries can be directed to the corresponding authors.

## Supplementary Materials

The following supporting information can be downloaded at: https://www.mdpi.com/article/10.3390/ma17225452/s1, Table S1: The limit value for metal concentrations in SS for Romanian and EPA legislations; Table S2: Distribution of toxic metals in fractions available for the environment corresponding to S1. Table S3: Distribution of toxic metals in fractions available for the environment corresponding to S2. Table S4: Distribution of toxic metals in fractions available for the environment corresponding to S3. Table S5: Distribution of toxic metals in fractions available for the environment corresponding to S4. Table S6: Distribution of toxic metals in fractions available for the environment corresponding to S5. Table S7: The leachable content of the studied S1–S5 of metal cations to the environment.
