# Supplementary material for: Ultrasound-Assisted Community Bureau of Reference (BCR) Procedure for Heavy Metal Removal in Sewage Sludge
Source: Materials (Basel). 2024 Nov 8;17(22):5452. doi: 10.3390/ma17225452 (PMC11595544; doi:10.3390/ma17225452)
Supplement: Supplementary file 1 [file materials-17-05452-s001.zip › materials-3280355-supplementary.pdf]

materials

MDPI

Supplementary Material

Ultrasound-Assisted Community Bureau of Reference (BCR) Procedure for Heavy Metal Removal in Sewage Sludge

Nicoleta Mirela Marin [1,2,3], Toma Galaon [1,3,*] and Luoana Florentina Pascu [1,*]

[1] National Research and Development Institute for Industrial Ecology ECOIND, Street Podu Dambovitei No. 57-73, District 6, 060652 Bucharest, Romania; nicoleta.marin@incdecoind.ro
[2] Department of Oxide Materials Science and Engineering, National University of Science and Technology Politehnica Bucharest, 1–7 Gh. Polizu, 060042 Bucharest, Romania
[3] Department of Analytical and Physical Chemistry, University of Bucharest, 4-12 Regina Elisabeta Bd., 030018 Bucharest, Romania
* Correspondence: tomagalaon@yahoo.com (T.G.); luoanapascu@yahoo.com (L.F.P.)

Table S1. The limit value for metal concentrations in SS for Romanian and EPA legislations.

Metals	As	Cd	Cr	Co	Cu	Mo	Ni	Pb	Sb	Zn
Inert waste *	0.5	0.04	0.5	-	2	0.5	0.4	0.5	0.06	4
Non-hazardous waste *	2	1	10	-	50	10	10	10	0.7	50
Hazardous waste *	25	5	70	-	100	30	40	50	5	200
Romanian legislation **	10	10	500	50	500	-	100	300	-	2000
EPA ***	41	39	-	*	1500	-	420	300	-	2800

* The maximum values accepted according to order 95/2005 regarding "Establishment of acceptance criteria and preliminary procedures for accepting waste for storage [1]". ** Maximum admissible value accepted according to Romanian Order no. 344/2004 [2]. "-" values not imposed by the legislation. *** Maximum admissible value imposed by EPA [3].

Table S2. Distribution of toxic metals in fractions available for the environment corresponding to S1.

Metal (mg/Kg)	Exchangeable and Acid Soluble (F1)	Reducible (F2)	Oxidable (F3)	Residual (F4)	Sum of Fractions	Aqua Regia
As	0.56±0.11	0.31±0.07	0.07±0.03	3.39±1.01	4.33±1.22	4.5±0.75
Cd	0.09±0.66	0.12±0.05	0.05±0.02	0.69±0.15	0.95±0.88	0.95±0.54
Co	0.86±0.05	0.40±0.1	0.34±0.11	4.78±0.93	6.38±1.19	6.75±1.22
Cr	1.07±0.24	1.64±0.50	1.90±0.39	90.9±1.12	95.5±2.25	98.8±1.78
Cu	0.81±0.18	4.50±0.96	3.83±0.71	135±3.21	144±5.06	145±1.43
Mo	0.02±0.01	0.12±0.04	0.07±0.02	4.1±0.75	4.31±0.82	4.95±1.35
Ni	5.69±0.58	1.97±0.55	1.58±0.92	24.5±1.61	33.7±3.66	34.8±4.05
Pb	<0.07	1.10±0.43	0.91±0.12	29.3±0.72	31.3±1.27	31.8±4.4
Sb	0.01±0.01	<0.04	0.41±0.09	4.7±0.69	5.12±0.79	5.15±0.86
Zn	109±1.07	59.9±1.3	59.2±0.80	508±1.76	736±4.93	771±6.3

Table S3. Distribution of toxic metals in fractions available for the environment corresponding to S2.

Metal (mg/Kg)	Exchangeable and Acid Soluble (F1)	Reducible (F2)	Oxidable (F3)	Residual (F4)	Sum of Fractions	Aqua Regia
As	0.87±0.29	0.38±0.11	0.99±0.23	6.15±1.08	8.39±1.71	8.4±0.64
Cd	0.32±0.09	0.13±0.08	0.23±0.05	0.33±0.1	1.01±0.32	1.10±0.7
Co	0.48±0.11	1.87±0.44	2.72±0.66	5.81±1.07	10.9±2.28	10.45±2.3
Cr	0.64±0.27	0.80±0.19	1.25±0.25	36.4±2.25	39.1±2.96	40.2±3.4
Cu	4.26±0.36	3.65±0.10	7.74±1.04	83.6±1.11	99.3±2.61	109±2.17
Mo	<0.02	<0.02	<0.02	4.24±0.65	4.24±0.65	4.9±0.8
Ni	3.61±1.06	1.80±0.45	3.73±0.99	21.6±1.33	30.7±3.83	31.6±1.2
Pb	<0.07	1.22±0.56	2.42±0.70	30.0±2.15	33.6±3.41	34.1±4.1
Sb	0.12±0.03	0.02±0.01	0.32±0.05	3.16±0.94	3.62±1.03	4.15±0.97
Zn	140±1.27	46.3±3.5	115±2.55	109±2.95	410±9.97	455±2.7

Table S4. Distribution of toxic metals in fractions available for the environment corresponding to S3.

Metal (mg/Kg)	Exchangeable and Acid Soluble (F1)	Reducible (F2)	Oxidable (F3)	Residual (F4)	Sum of Fractions	Aqua Regia
As	0.51±0.08	0.12±0.35	0.62±0.07	<0.05	1.25±1.75	1.18±0.02
Cd	0.28±0.1	0.32±.0.12	2.92±0.33	8.14±0.61	11.7±1.16	12±0.59
Co	1.08±0.11	0.14±.0.04	2.11±0.37	3.9±0.48	7.23±1.00	7.35±0.12
Cr	3.51±0.85	0.69±0.12	62.7±1.02	214±8.83	281±10.82	299±6.02
Cu	4.13±0.44	1.42±0.30	37.3±1.64	263±4.92	306±7.30	321±3.74
Mo	0.56±0.15	0.08±0.01	0.14±0.02	354±4.11	355±4.29	349±10.3
Ni	6.61±0.17	0.6±0.25	8.83±0.15	17.9±0.92	33.9±1.49	35±2.16
Pb	<0.07	0.51±0.07	29.6±0.91	3.12±0.50	33.2±1.48	34.3±0.7
Sb	0.50±0.19	0.04±0.01	2.85±0.13	6.08±0.56	9.47±0.89	9.66±0.29
Zn	349±4.97	73±3.74	233±4.92	2058±8.73	2713±22.36	2722±8.49

Table S5. Distribution of toxic metals in fractions available for the environment corresponding to S4.

Metal (mg/Kg)	Exchangeable and Acid Soluble (F1)	Reducible (F2)	Oxidable (F3)	Residual (F4)	Sum of Fractions	Aqua Regia
As	0.55±0.06	0.05±0.02	0.12±0.05	1.34±0.08	2.06±0.21	2.52±0.21
Cd	0.12±0.02	0.09±0.05	0.01±0.01	0.69±0.05	0.91±0.13	0.99±0.39
Co	1.17±0.10	0.25±0.06	0.13±0.02	3.2±0.34	4.75±0.52	5.14±0.16
Cr	0.48±0.02	0.23±0.03	1.37±0.10	27.1±0.62	29.2±0.77	30.4±1.11
Cu	0.8±0.07	2.61±0.01	1.58±0.20	112±2.87	117±3.15	123±1.25
Mo	<0.02	0.08±0.43	0.03±0.02	4.94±0.08	5.06±0.53	5.14±0.07
Ni	7.08±0.28	1.24±0.11	0.72±0.05	14.9±0.80	23.9±1.24	24.2±1.64
Pb	<0.07	0.35±0.04	0.9±0.10	1.12±0.05	2.37±0.19	2.50±0.14
Sb	0.12±0.03	0.05±0.01	0.13±0.06	1.9±0.22	2.20±0.32	2.34±0.25
Zn	173±2.87	44.3±0.24	4.22±0.13	207±1.25	429±4.49	440±8.5

Table S6. Distribution of toxic metals in fractions available for the environment corresponding to S5.

Metal (mg/Kg)	Exchangeable and Acid Soluble (F1)	Reducible (F2)	Oxidable (F3)	Residual (F4)	Sum of Fractions	Aqua Regia
As	<0.05	0.05±0.03	<0.05	5.01±1.25	5.06±1.28	5.20±1.64
Cd	0.03±0.02	0.02±0.14	0.12±0.08	0.01±0.02	0.18±0.26	0.22±0.04
Co	0.85±0.32	0.64±0.06	3.43±0.24	6.86±0.98	11.8±1.60	12.6±2.93
Cr	0.89±0.27	0.34±0.02	40.4±2.04	104±2.49	146±4.82	151±17.1
Cu	11.4±0.48	4.39±0.29	85.7±1.8	176±12.3	277±14.87	290±12.5
Mo	<0.02	0.05±0.44	0.08±0.49	5.71±0.42	5.84±1.35	5.99±1.25
Ni	1.86±0.37	0.69±0.13	10.1±2.13	18.7±2.12	31.4±4.75	32±0.94
Pb	<0.07	<0.07	<0.07	11.4±1.91	11.4±1.91	12.1±1.2
Sb	0.11±0.04	0.04±0.46	2.38±0.22	7.84±0.88	10.4±1.60	10.9±1.73
Zn	5.58±0.26	1.07±0.09	4.86±0.14	41.8±1.72	53±2.21	54±1.3

Table S7. The leachable content of the studied S1-5 of metal cations to the environment.

Metal (mg/kg)	S1	S2	S3	S4	S5
As	0.27±0.040	0.21±0.037	0.04±0.012	0.21±0.016	0.07±0.009
Cd	0.01±0.004	0.04±0.022	0.02±0.009	0.07±0.009	0.05±0.008
Co	0.13±0.025	0.04±0.009	0.32±0.116	0.07±0.017	0.01±0.012
Cr	0.06±0.033	0.03±0.008	0.07±0.012	0.01±0.001	<0.01
Cu	1.21±0.045	0.40±0.111	20±2.828	0.54±0.041	0.12±0.050
Mo	0.89±0.128	0.25±0.033	0.85±0.071	0.58±0.046	0.02±0.001
Ni	1.2±0.170	0.03±0.008	1.80±0.249	0.3±0.017	0.8±0.026
Pb	0.05±0.026	0.02±0.009	0.04±0.012	0.01±0.005	0.02±0.002
Sb	0.02±0.017	0.01±0.008	0.01±0.004	0.01±0.001	0.01±0.001
Zn	0.81±0.078	0.36±0.105	1.70±0.163	0.80±0.071	0.2±0.021

The value notated with < in Table S2–S6 represent results situated below the determination limit of analytical method.

References

1. Romanian Order no. 95/2005 Establishing Acceptance Criteria and Preliminary Procedures for Acceptances of Waste Storage and National List of Waste Accepted in Each Class of Landfill. Available online: https://legislatie.just.ro/Public/DetaliiDocument/59751 (accessed on 1 September 2024).
2. Romanian Order no. 344/2004 Regarding Technical Rules on Environmental and in Particular, Soils When Are Used Amended Sewage Sludge in Agriculture. Available online: https://legislatie.just.ro/Public/DetaliiDocument/55968 (accessed on 1 September 2024).
3. USEPA. Standards for the Use or Disposal of Sewage Sludge, 40 C.F.R. Sect. 503. US Environmental Office of Wastewater Management. 1993. Available online: https://p2infohouse.org/ref/48/47618.pdf (accessed on 1 September 2024).

Disclaimer/Publisher's Note: The statements, opinions and data contained in all publications are solely those of the individual author(s) and contributor(s) and not of MDPI and/or the editor(s). MDPI and/or the editor(s) disclaim responsibility for any injury to people or property resulting from any ideas, methods, instructions or products referred to in the content.
